# A Lightweight Network for Accurate Coronary Artery Segmentation Using X-Ray Angiograms

**DOI:** 10.3389/fpubh.2022.892418

**Published:** 2022-05-25

**Authors:** Xingxiang Tao, Hao Dang, Xiaoguang Zhou, Xiangdong Xu, Danqun Xiong

**Affiliations:** ^1^School of Modern Posts/Automation, Beijing University of Posts and Telecommunications, Beijing, China; ^2^School of Information Technology, Henan University of Chinese Medicine, Zhengzhou, China; ^3^Department of Cardiology, Jiading District Central Hospital Affiliated Shanghai University of Medical and Health Sciences, Shanghai, China

**Keywords:** deep learning, lightweight networks, coronary arteries segmentation, image processing, attention mechanism

## Abstract

An accurate and automated segmentation of coronary arteries in X-ray angiograms is essential for cardiologists to diagnose coronary artery disease in clinics. The existing deep learning-based coronary arteries segmentation models focus on using complex networks to improve the accuracy of segmentation while ignoring the computational cost. However, performing such segmentation networks requires a high-performance device with a powerful GPU and a high bandwidth memory. To address this issue, in this study, a lightweight deep learning network is developed for a better balance between computational cost and segmentation accuracy. We have made two efforts in designing the network. On the one hand, we adopt bottleneck residual blocks to replace the internal components in the encoder and decoder of the traditional U-Net to make the network more lightweight. On the other hand, we embed the two attention modules to model long-range dependencies in spatial and channel dimensions for the accuracy of segmentation. In addition, we employ Top-hat transforms and contrast-limited adaptive histogram equalization (CLAHE) as the pre-processing strategy to enhance the coronary arteries to further improve the accuracy. Experimental evaluations conducted on the coronary angiograms dataset show that the proposed lightweight network performs well for accurate coronary artery segmentation, achieving the sensitivity, specificity, accuracy, and area under the curve (AUC) of 0.8770, 0.9789, 0.9729, and 0.9910, respectively. It is noteworthy that the proposed network contains only 0.75 M of parameters, which achieves the best performance by the comparative experiments with popular segmentation networks (such as U-Net with 31.04 M of parameters). Experimental results demonstrate that our network can achieve better performance with an extremely low number of parameters. Furthermore, the generalization experiments indicate that our network can accurately segment coronary angiograms from other coronary angiograms' databases, which demonstrates the strong generalization and robustness of our network.

## Introduction

**Figure 1:**
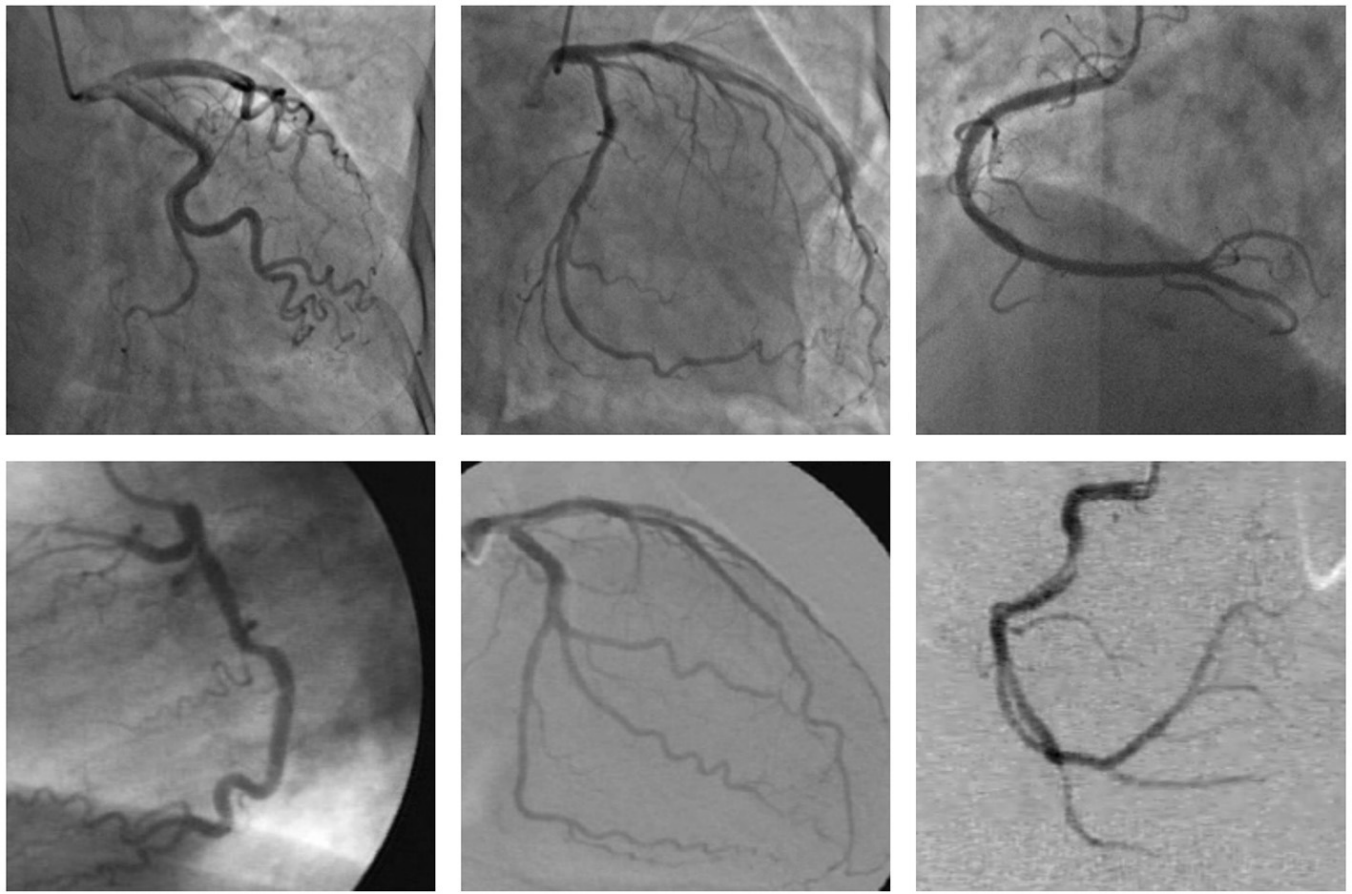
These examples of the X-ray angiogram image with nonuniform illumination, low contrast ratios, and low signal-to-noise ratios. The images in the first row are selected from the clinical coronary angiograms dataset, while the second row of the data is from the Database X-ray Coronary Angiograms.

Cardiovascular diseases are the primary cause of death worldwide (1), representing 32% of all global deaths in 2019 (2), and coronary artery disease (CAD) is the most common cardiovascular disease (3). Moreover, more than three-quarters of deaths due to cardiovascular disease occur in the low- and middle-income countries (2) since these countries lack experienced and knowledgeable experts, as well as advanced diagnostic technologies (4). CAD is caused by too much plaque buildup inside the arteries that supply oxygen-rich blood to the heart, thus leading to a narrowing or blockage of the arteries. A narrowed or blocked coronary artery could result in a heart attack, stroke, or sudden cardiac death (5). Among the many existing diagnoses of CAD, coronary angiogram is the most common in the clinic performed using an X-ray to observe the morphology of the arteries through the injection of a liquid contrast agent (6), and the X-ray coronary angiogram is the gold standard for diagnosing coronary artery disease (7). Accurate segmentation of the vessel structure of coronary arteries is a vital step in the correct diagnosis of CAD. An accurate segmentation of the coronary arteries can reveal the stenosis of the vessel clearly, which can provide the basis for the quantification and assessment of vascular stenosis. However, the quality of the X-ray angiogram is not good due to nonuniform illumination, low contrast ratios, low signal-to-noise ratios, the presence of other tissues, and camera motion as shown in Figure 1. The low quality of the X-ray angiogram makes manual segmentation of vessels by cardiologists in the diagnosis of CAD a time-consuming and challenging task. To improve the efficiency of the diagnosis of CAD, automated and accurate segmentation of coronary arteries is necessary.

To solve the above challenging issue, many excellent segmentation approaches have been proposed. These approaches can commonly be categorized into model-based methods, pattern-recognition methods, tracking-based methods (8, 9), and deep-learning methods. Here, we broadly classify the methods above into traditional methods and deep-learning methods. In Zhao et al. (10) and Zhao et al. (11), region-based active contours are proposed to segment vessels. The key to the method is to detect the boundaries of the vessels by curves or surfaces. In Mendonca et al. (12), an approach combined with the detection of centerlines and morphological reconstruction is developed to extract the retinal blood vessels. In Carrillo et al. (13), recursive tracking is designed to detect vascular trees in 3D medical images. In Yureidini et al. (14), vasculature tracking is utilized to segment vessels in 3D rotational angiography. However, these traditional segmentation methods might be not effective in segmenting X-ray angiograms with low quality.

Deep learning has been widely used in the field of medical image processing, which contains image recognition and segmentation. Moreover, plenty of methods based on deep learning have been proposed to segment vessels. For instance, Yan et al. (15) constructed a deep learning model combined with segment-level and pixel-wise losses for retinal vessel segmentation. In (16–18), these coronary artery segmentation methods are all developed based on deep learning. In all the deep learning methods above, the convolutional neural network (CNN) is utilized to filter features. To improve the performance of the network, a general way is to make the networks (19, 20) deeper and more complicated. In Jiang et al. (18) and Zhu et al. (21), a multiscale fusion network was conducted to capture long-range dependencies in coronary artery segmentation tasks. Dilated convolutions (22) can also help to get more context information by expanding the receptive field. Recently, more and more researches (23, 24) have integrated the attention mechanism into their network to capture contextual information. All these approaches can improve the performance of the network significantly. However, employing these methods above has been achieved at the expense of the scale and efficiency of the network. For assistant diagnosis of CAD to become widespread in the regions where there is lack of medical resources (experienced and knowledgeable experts, as well as advanced medical equipment), it is necessary to deploy the automatic segmentation of coronary angiography algorithm on affordable mobile devices for application in the clinic.

To address the problems above, a novel network architecture based on bottleneck residual blocks (25) and lightweight attention mechanisms (26) is proposed in this study for the segmentation of coronary arteries. In detail, we employed bottleneck residual blocks to replace the components from the regular U-Net (27) with some subtle adjustments. The bottleneck residual block is mainly constituted by depthwise separable convolutions (28, 29) to reduce the computation. To further improve the performance of our network, we develop a patch attention module to aggregate long-range contextual information, which can model the relations between each patch of the coronary angiogram. Furthermore, the proposed the attention module that not only improves the network performance but also minimizes the computational cost. To establish more connections between feature maps channels, we adopted squeeze and excitation (SE) blocks (23) to assign weights between channels. The SE block is also lightweight and hardly burdens our network. Therefore, we designed the network with an optimal trade-off between performance and lightweight.

The main contributions of our work are as follows:

1. The bottleneck residual blocks are integrated into the regular U-Net to make our network more lightweight.
2. A novel attention module, named patch attention module, is proposed to model the spatial dependencies of the coronary angiogram with low computational cost.
3. The SE blocks are embedded into the network to establish more connections between feature maps channels.
4. A small database of coronary angiography is established to promote our scientific research.

The remaining part of this paper is organized as follows: Section Related Work presents the related work of our method, Section Methodology specifically introduces the method of our work, Section Experiments and Results Analysis shows the ablation studies and the corresponding results, Section Discussion provides discussions about the proposed method, and Section Conclusion gives the conclusion of our work.

## Related Work

In the following, we review recent advances in three aspects: Section Lightweight convolutional neural networks, Section Semantic segmentation in vessels images, and Section Attention mechanism.

### Lightweight Convolutional Neural Networks

Recently, many small and efficient networks have been proposed for computer vision tasks. Some of the networks can be classified as only shrinking the network size, but not caring about speed. A novel inverted bottleneck structure, which is primarily constituted by depthwise separable convolutions (28, 29) and pointwise convolutions, has been developed in MobileNets (25, 30, 31) to reduce the computation. This structure can reduce the size while reducing the computation, thus increasing the inference speed of networks. ShuffleNets (32, 33) simplify pointwise convolutions to make networks lightweight by employing the group convolutions and channel's shuffle. It is crucial to gain the factorized convolutions using the small networks in the above methods (30). Distillation (34) is another approach to train small networks, and a larger network is employed to teach the small networks. Shuvo et al. (35) proposed a novel lightweight architecture for multimodal Biomedical Image Segmentation by modifying the structure of the standard U-Net. In our work, we mainly utilized the ideas from MobileNets to make our network more lightweight.

### Semantic Segmentation in Vessels Images

As is well known, more and more networks based on deep convolutional neural networks have been employed to address medical image semantic segmentation (36) tasks. In the field of vessels segmentation, deep learning is also widely used. In (37), an edge-aware flowing into U-Net encoder-decoder architecture was presented to guide the retinal vessel segmentation. In (38), an attention–inception-based U-Net was proposed for retinal vessel segmentation. In (39), a fully convolutional multichannel network was proposed to segment the coronary angiograms. In (40), a modified version of U-Net was developed for retinal blood vessel segmentation. A convolution neural network with a reinforcement sample learning strategy was utilized for retinal vessel detection in (41). A complex network combined a convolutional neural network and conditional random field layers to segment retinal vessels in (42). All these approaches above increased the depth and width of the network to improve its performance. However, the deeper and wider networks represent more parameters and more computation. In clinically assisted diagnosis, it is important that more accurate and fast segmentation networks, as well as more convenient mobile devices are available to our doctors. To address the problem above, we replaced almost all components of the traditional U-Net (27) with bottleneck residual blocks (25). The bottleneck residual blocks can reduce numerous parameters and computations for our network.

### Attention Mechanism

Attention mechanism originates from the exploration of human vision, and it has been widely applied in many tasks of computer vision (23, 43, 44). In recent years, the ability of deep neural networks to process information is becoming stronger and stronger, but the computation ability is still the bottleneck of the development of the neural network. The attention mechanism can focus on the key regions of the task and suppress irrelevant information so as to obtain a better information processing effect with the same amount of computing resources. The attention mechanism is always employed to model long-range dependencies in spatial and channel dimensions in image tasks. In SENet (23), the attention mechanism in channel-wise is utilized to promote channels of feature maps that are useful to the task and suppress channels that are not useful to the task. DANet (24) introduces two parallel attention modules, a position attention module and a channel attention module, to capture feature dependencies in the spatial and channel dimensions, respectively. Yi et al. (45) presented a semantic segmentation network with a channel-coordinate attention feature fusion module to improve the network performance. In this work, we employed the SE block from SENet (23) to be embedded into the bottleneck residual block to capture feature dependencies in the channel-wise. In addition, we also modeled the spatial long-range dependencies in our network by modifying the position attention module from DANet (24).

## Methodology

**Figure 2:**
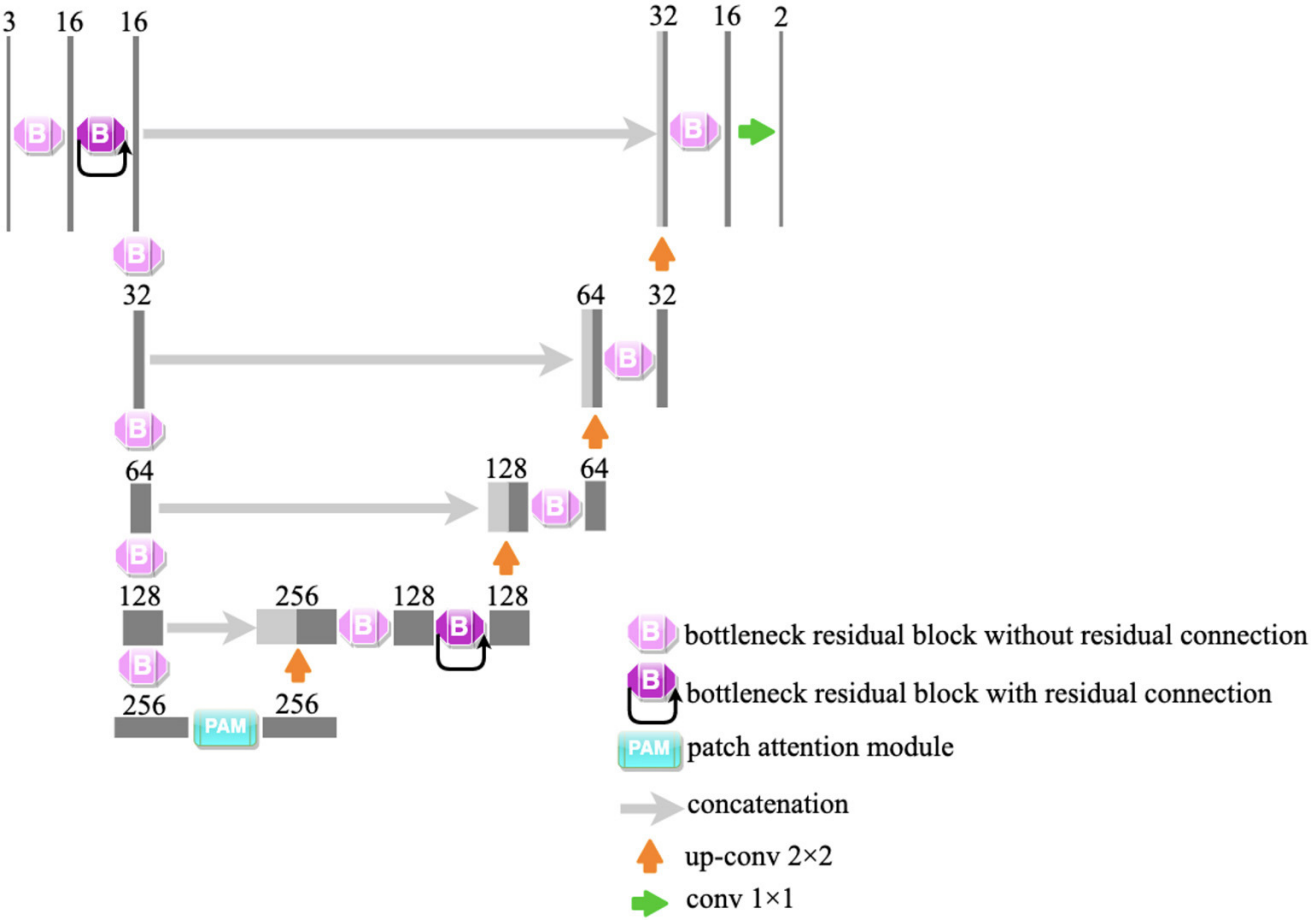
The illustration of the proposed network. We adopt bottleneck residual blocks to replace the internal components of traditional U-Net. The architecture of the bottleneck residual block is shown in Figure 6. PAM is an attention module and is shown in Figure 7.

In our study, a lightweight segmentation network, named Bottleneck Residual U-Net (BRU-Net), is proposed for coronary arteries segmentation in X-ray angiograms. The BRU-Net is a variant of the U-Net model (27), and we adopted bottleneck residual blocks (25) to replace the internal components in the encoder and decoder of traditional U-Net (27) to make the network more lightweight. Figure 2 shows the architecture of the proposed network. The details of the proposed framework are mentioned in the following.

### Datasets

Database X-ray coronary angiograms (46) (DCA1), which originate from the Cardiology Department of the Mexican Social Security Institute, UMAE T1-León, and the clinical coronary angiograms (CCA) dataset (from the collaborating hospital) are utilized in our experiments to evaluate our proposed BRU-Net.

The DCA1 dataset consists of 134 X-ray coronary angiograms and the corresponding ground-truth images formed by an expert cardiologist. Each angiogram is 300 × 300 pixels (46) and we cropped them to 288 × 288 in our work. We randomly selected 104 angiograms as the training set and the rest as the test set.

The CCA dataset contains 150 X-ray coronary angiograms and the manual ground-truth images were outlined by experts from the collaborating hospital. The resolution of each angiogram is 768 × 768 and we cropped the angiograms to 576 × 576. A total of 130 angiograms are divided into a training set, and the remaining 20 angiograms are taken into the test set. In our work, we employed random rotation, random crop, random vertical flip, and random horizontal flip as the augmentation strategies to augment the training dataset.

**Figure 3:**
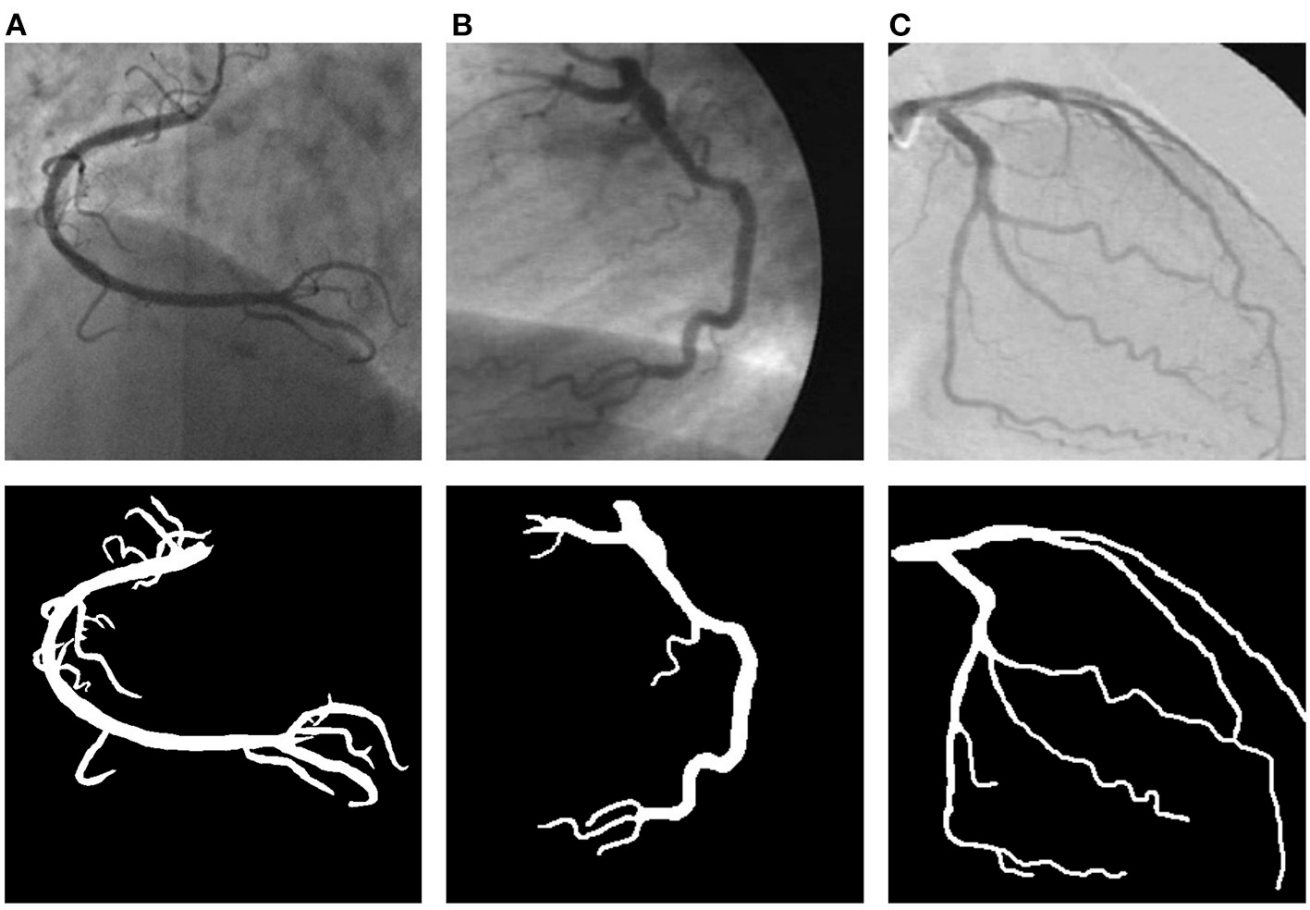
The original angiograms and corresponding labels from the two datasets. The images in the first row are the original angiograms, while the corresponding labels are in the second row. The images in **(A)** column are from CCA, and the images in **(B)** and **(C)** are from DCA1.

The angiograms are very complex due to the problems such as nonuniform illumination, low contrast ratios, low signal-to-noise ratios, the presence of other tissues, and camera motion (16, 47). Moreover, the angiograms from the CCA dataset are much more complex than the angiograms from DCA1 dataset. We show some examples of original angiograms and corresponding labels from the two datasets in Figure 3.

### Image Pre-processing

**Figure 4:**
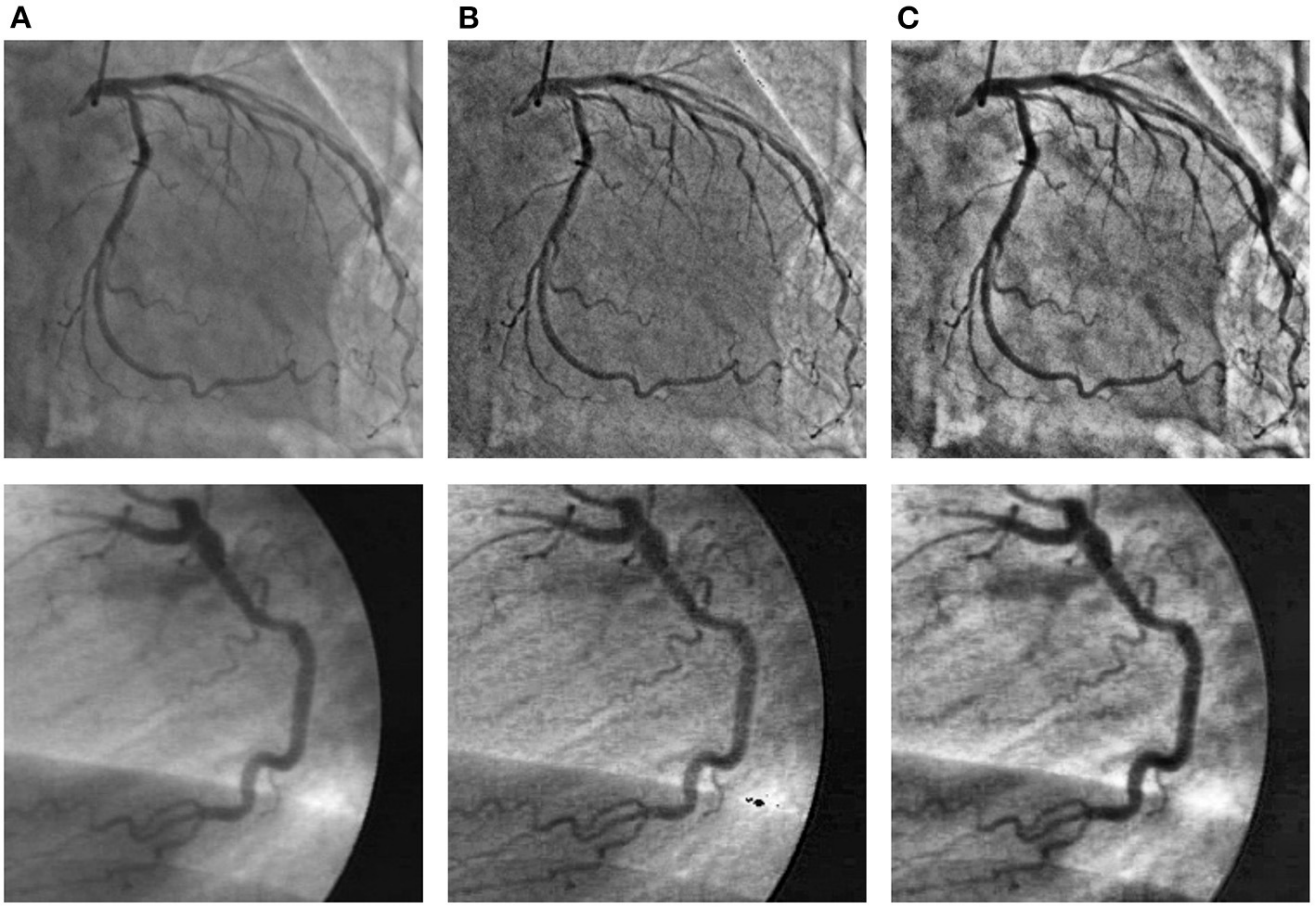
The angiograms are pre-processed by the two strategies. The first column **(A)**: original angiograms; the second column **(B)**: angiograms after Top-hat transform; the third column **(C)**: angiograms after CLAHE.

To improve the problems of nonuniform illumination and low contrast ratios in angiograms, we adopted some image pre-processing strategies. We utilized two pre-processing strategies to enhance the angiograms, namely top-hat transform (48, 49) and contrast-limited adaptive histogram equalization (CLAHE) method (50), respectively. We show sample angiograms preprocessed by the two strategies in Figure 4.

**Figure 5:**
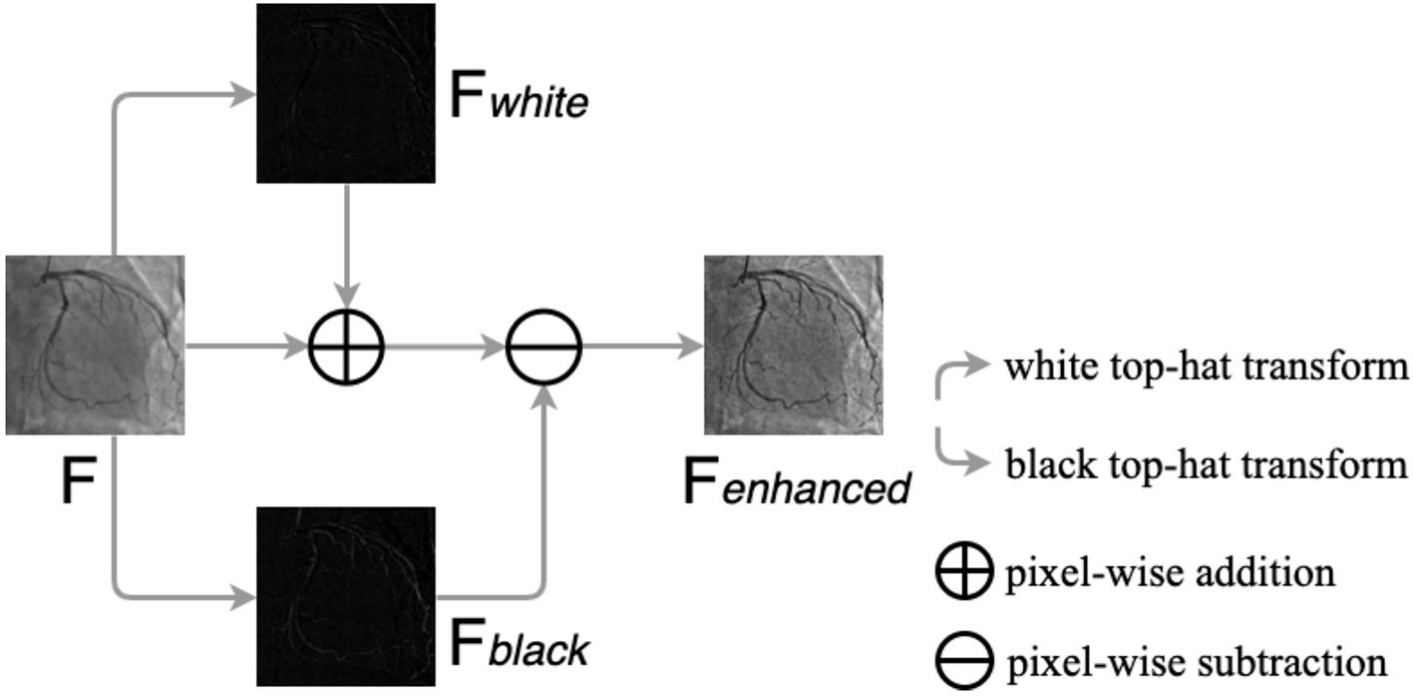
The procedure of the top-hat transforms. *F* is the original gray angiogram image, *F*_*white*_ and *F*_*black*_ are obtained by white and black top-hat transform, respectively. *F*_*enhanced*_ is the enhanced angiogram image.

Top-hat transform is a mathematical morphological operation that extracts small elements and details from given images (48, 49). The white top-hat and the black top-hat are the two types of top-hat transforms. In the white top-hat, the bright regions in the image are brighter than their surroundings, while the dark areas are highlighted in the black top-hat. Inspired by Nasr-Esfahani et al. (51), we adjusted nonuniform lighting conditions and enhanced the contrast of the angiograms by the top-hat transform procedure, which is illustrated in Figure 5. The procedure contains three main steps: converting the original angiogram to the gray one *F*, transforming *F* to *F*_*white*_ and *F*_*black*_ by white top-hat transform and black top-hat transform, respectively, and adding *F*_*white*_ to *F* pixel in pixel and subtracting *F*_*black*_ pixel in pixel. After the procedure above, we can gain *F*_*enhanced*_ with increased contrast.

To further enhance the contrast of the angiogram images, we employed the CLAHE method. The CLAHE method can increase the overall region contrast of the angiogram image, while reducing the problem of noise amplification by limiting the contrast amplification (50).

### Bottleneck Residual Block

**Figure 6:**
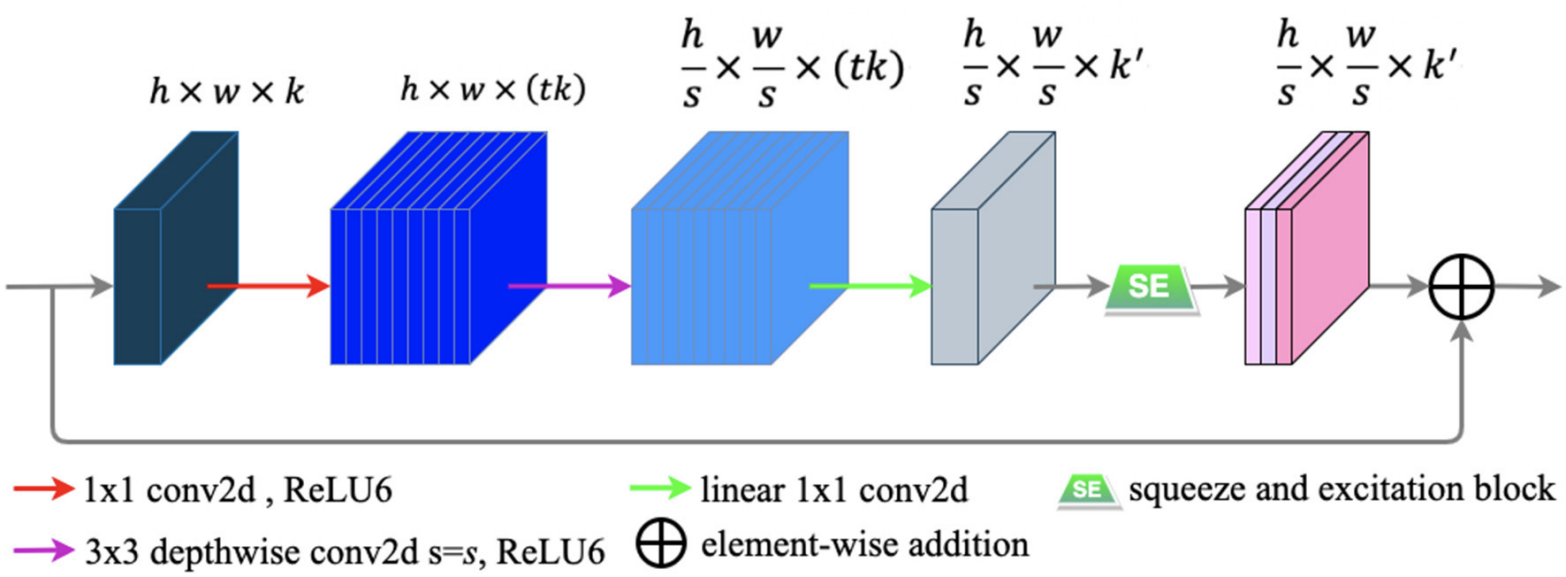
The bottleneck residual block transforming from *k* to *k*′ channels, with stride *s*, and expansion factor *t* (default value is 2). The residual connection is only used when *s* = 1 *and k* = *k*′.

A novel convolutional neural network layer has been developed by MobileNetV2 (25), which achieves high performance in mobile and embedded vision applications. The essential component of the novel layer is the bottleneck residual block (25), which contains inverted residual and linear bottleneck structures. The bottleneck residual block is portrayed in Figure 6.

The bottleneck residual block has three main following steps to process an input feature map *F* ∈ ℝ^*H* × *W* × *C*^. First, to expand the low-dimensional *F* ∈ ℝ^*h* × *w* × *k*^ to a higher-dimensional *F* ∈ ℝ^*h* × *w* × (*tk*)^ by using a 1 × 1convolution called pointwise convolution (25). Next, a 3 × 3 depthwise separable convolution is performed as a filter that features an operation to produce a new representation $F\in {\mathbb{R}}^{\frac{h}{s}\times \frac{w}{s}\times \left(tk\right)}$. Finally, a pointwise convolution is used to project the spatially filtered feature map to a low-dimensional subspace. In our work, we modified the bottleneck block by embedding the SE block into it to capture the feature-dependencies in the channel-wise. The SE block is illustrated in the subsection E of this section. Each of the first two steps is followed by batch normalization (29) and ReLU6 activation (30), and only the last step is followed by batch normalization. The formulas of ReLU6 and ReLU (52) are defined as follows:


(1) $$\begin{array}{l} ReLU6(x)=\text{min}(\max\left(0,x\right),6) \end{array}$$

(2) $$\begin{array}{l} ReLU(x)=\max\left(0,x\right) \end{array}$$

ReLU6 is like the well-known ReLU (52), but it is more robust when used with low-precision computation (30).

In our work, we utilized the bottleneck residual blocks for the regular convolutions in U-Net to reduce computational cost to take place. In the network, we employed the original bottleneck residual blocks without embedding the SE blocks.

### Patch Attention Module

Most modern methods consider the semantic segmentation task as a dense prediction task. However, an incorrect prediction will lead to inaccurate segmentation results, especially the image with a complex background. The problem above is mainly due to the lack of remote context information. To model rich context relationships on local feature representation, Fu et al. (24) introduced the position attention module. The position attention module encodes a wider range of context information into local features to improve their representational capabilities (24).

**Figure 7:**
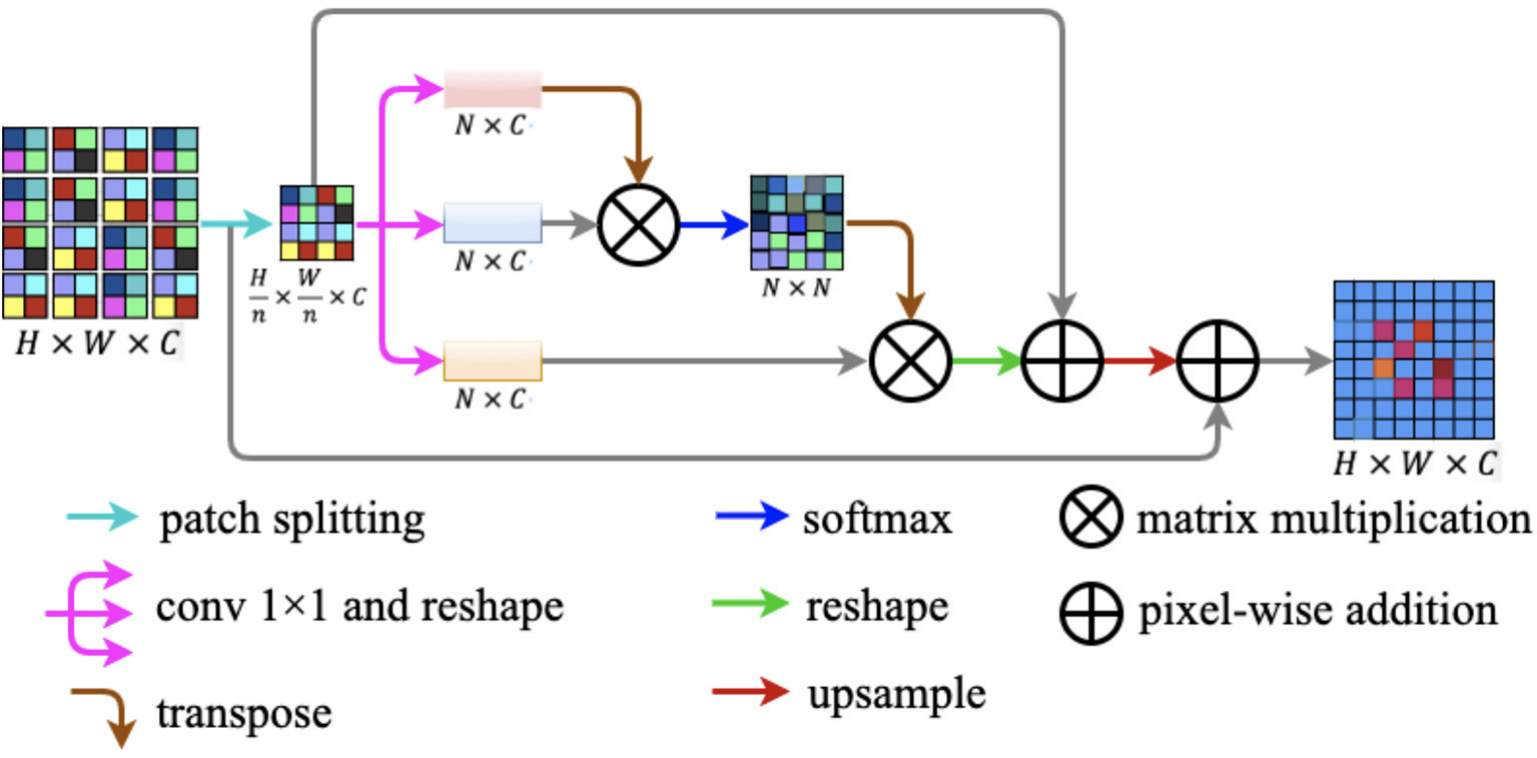
Patch attention module. It consists of two modules: a patch splitting module and an attention module.

In this study, we design a novel lightweight attention module named patch attention module (PAM). PAM is developed to assist networks to capture remote context information while taking into account the lightness of the model. The patch attention module consists of two modules: a patch splitting module and an attention module. The structure diagram of our proposed patch attention module is illustrated in Figure 7.

To simultaneously address the problem of modeling spatial long-range dependencies and make the model lightweight, we utilized the relations between each patch of the feature map to replace the relations between the positions of each pixel. For an input feature map *F* ∈ ℝ^*H* × *W* × *C*^, we fed *F* into the patch-splitting module where *F* will be split into non-overlapping patches. Patch size is denoted by *n* × *n*, where *n* is a multiple of two. We regarded each patch as a position and formed a feature map with patches replaced by pixels. After the operation, we got a new feature map $F^{\prime }\in {\mathbb{R}}^{\frac{H}{n}\times \frac{W}{n}\times C}$.

After completing the above patch splitting operation, we fed the new feature map to the attention module. Firstly, $F^{\prime }\in {\mathbb{R}}^{\frac{H}{n}\times \frac{W}{n}\times C}\;$is fed into a parallel structure with three convolution layers to generate three new feature maps *q*(*F*′), *k*(*F*′), and *v*(*F*′) $\in {\mathbb{R}}^{\frac{H}{n}\times \frac{W}{n}\times C}$. Secondly, we reshaped the three new feature maps to ℝ^*N* × *C*^, where $N=\frac{H}{n}\times \frac{W}{n}$ is the amount of all the patches. Next, we transposed *q*(*F*′) to ℝ^*C* × *N*^ to perform matrix multiplication with *k*(*F*′) ∈ ℝ^*N* × *C*^ and then we obtained the patch spatial attention map *S* ∈ ℝ^*N* × *N*^ by a softmax layer. The interaction of each patch in the original feature map is defined by the following formula:


(3) $$\begin{array}{l} s_{mn}=\frac{exp\left({q\left(F^{\prime }\right)}_{m}•{k\left(F^{\prime }\right)}_{n}\right)}{\sum_{n=1}^{N}exp\left({q\left(F^{\prime }\right)}_{m}•{k\left(F^{\prime }\right)}_{n}\right)} \end{array}$$

where *s*_*mn*_ reflects the impact between the two patches, and the more similar the two patches are the larger is *the s*_*mn*_. After the above procedures, we implemented a matrix multiplication between transformed *S* and *v*(*F*′). After this, we reshaped *S* and summed α × *S* and *F*′ in an element-wise manner, where α is a learnable scale parameter. Finally, we up-sampled the result generated above to ℝ^*H* × *W* × *C*^ and projected the patch spatial attention to the original feature map *F* ∈ ℝ^*H* × *W* × *C*^ by implementing a pixel-wise addition between the up-sampled result and the original feature map *F*.

In our work, we employed the patch spatial attention instead of the pixels position spatial attention to save computing costs and make our network more lightweight, and the space complexity is $\frac{1}{n^{4}}$ times the pixels' position spatial attention. The proposed patch attention module can be embedded into other networks expediently.

### Squeeze and Excitation Block

Recently, many researches (43, 44) have proved that spatial-wise attention, such as the patch attention module mentioned above, can improve the performance of the network obviously in semantic segmentation tasks. Moreover, embedding channel-wise attention module into the network can also yield positive results. The research (23) proposed a lightweight channel-wise attention block, namely the squeeze and excitation (SE) block. The essential feature of the SE block is to make the network exploit the beneficial features while suppressing the useless features in the channel-wise for the tasks (23).

**Figure 8:**
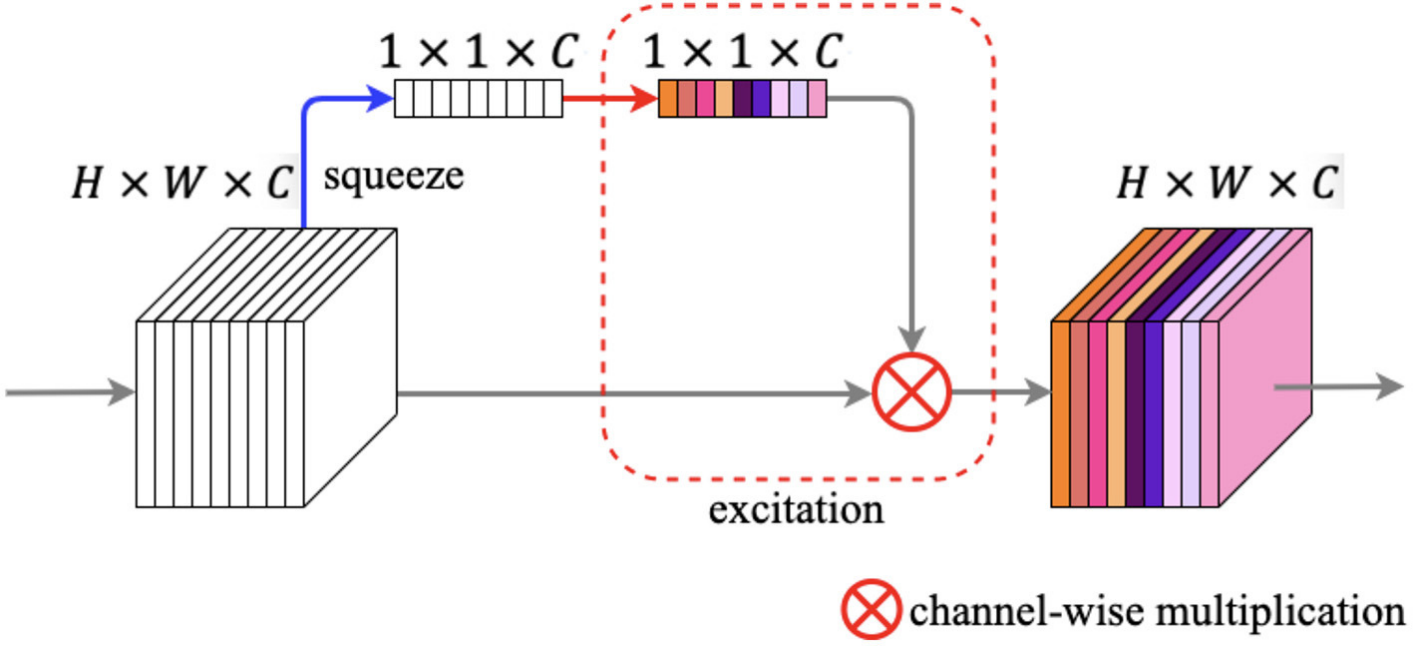
Squeeze and excitation block. The blue arrow denotes squeeze operation, while the red rounded rectangle denotes excitation operation.

The illustration of the squeeze and excitation block is shown in Figure 8. The SE block contains two primary components: squeeze operation and excitation operation. We feed an input feature map *F* ∈ ℝ^*H* × *W* × *C*^ into the SE block, and after the squeeze operation we got a vector *V* ∈ ℝ^1 × 1 × *C*^. The squeeze operation changes the spatial dimensions of the feature map from *H* × *W* to 1 × 1 in every channel by global average pooling. The calculation of each channel can be written as:


(4) $$\begin{array}{l} z=\frac{1}{H\times W}\overset{H}{\underset{i=1}{\sum }}\overset{W}{\underset{j=1}{\sum }}f\left(i,j\right) \end{array}$$

where *z* ∈ ℝ^*C*^ and *f* denotes every pixel of each channel. Next, the vector enters the excitation step and passes through a bottleneck structure with two fully connected (FC) layers, and after the first FC layer is a ReLU activation, which employs the sigmoid activation after the second FC layer. After the procedures above, we can get an output *s* ∈ ℝ^1 × 1 × *C*^, which denotes each channel weight. Finally, a channel-wise multiplication between the feature map *F* and *s* is used to zoom the spatial maps for each channel.

In our work, we embedded the SE block here into the proposed network to boost the capacity of our method with a low computational cost.

### Proposed Network

We employed the plain BRU-Net without any extra component as the backbone of our work. The bottleneck residual blocks are employed to perform a convolution operation on each channel separately to reduce the computation. To improve the performance of segmentation, we inserted the patch attention module after the encoder module and inserted the squeeze and excitation block (23) into the bottleneck residual blocks of plain BRU-Net as shown in Figure 2. As is known to all, the vessel regions occupy a very small portion of the overall angiography image, while most of it is the background. To solve the heavily unbalanced classes, we utilized a weighted cross-entropy loss (53) function, while employing dice loss (54) to coordinate with the cross-entropy loss in the training procedure. These loss functions can be defined as:


(5) $$\begin{array}{l} L_{WCE}\left(y,\hat{y}\right)=-(\alpha^{*}w^{*}y_{i}\log\left({\hat{y}}_{i}\right)+{\left(1-w\right)}^{*}(1-y_{i})\log\left(1-{\hat{y}}_{i}\right)) \end{array}$$

(6) $$\begin{array}{l} w=1-\frac{y}{H\times W} \end{array}$$

(7) $$\begin{array}{l} L_{Dice}\left(y,\;ŷ\right)=1-\frac{2yŷ}{y+ŷ} \end{array}$$

(8) $$\begin{array}{l} L=L_{WCE}+\beta^{*}L_{Dice} \end{array}$$

where the Equation 5 is weighted cross-entropy loss function, *y* and ŷ are the ground truth and the prediction result, respectively. The hyper-parameter α is a fine-tuning parameter, which we set to 0.2 empirically. The hyper-parameter *w* is the weight designed to alleviate category imbalance, and Equation 6 is the definition. Equation 7 is the dice loss function, and we finally adopted the loss function as defined in Equation 8 with a hyper-parameter β, setting it as 0.01.

## Experiments and Results Analysis

In this section, we first present the implementation details and the evaluation metrics, then evaluate the effectiveness of the approach proposed in this study by multiple ablation studies. We employ BRU-Net without any extra component, mentioned in Section Methodology, as the backbone architecture to implement all the comparative experiments in this section.

### Implementation Details

We implement our method based on Pytorch (55) and train it on one NVIDIA GeForce RTX 3090 GPU. We train the proposed network by exploiting the adaptive moment estimation (Adam) (56) optimization method with momentum 0.9, weight decay 0.0001, and batch size 16. Enlightened by the work of Chen et al. (22), we employed the poly learning rate policy, and the learning rate can be indicated as ${baselr\times \left(1-\frac{iter}{\max\text{\_}iter}\right)}^{0.9}$ with *lr* 4 × 10^−3^. The augmentation strategies are described in Section Introduction, and to be fair, we resize the images in the test set to 288 × 288 in all experiments.

### Evaluation Metrics

In our work, coronary angiograms segmentation is the task of segmenting the vessels from the background in X-ray angiograms. The specific process is to classify vessels pixels and background pixels correctly. We evaluated our approach on various evaluation metrics to measure the segmentation performance. Accuracy (ACC) is a crucial evaluation metric to appraise the classification task. In our work, the accuracy is the ratio of the sum of correctly classified vessel pixels and background pixels to all pixels. However, accuracy is not enough to evaluate the approach because of the unbalanced classes in the coronary angiographic images, where the number of the vessel pixels is far less than the number of the background pixels. Therefore, we also employed the other three metrics: sensitivity (SE), specificity (SP), and area under curve (AUC). Sensitivity and specificity can measure the capability of our network to detect correctly vessel pixels and background pixels, respectively. The above metrics' formulas are as follows:


(9) $$\begin{array}{l} ACC=\frac{TP+TN}{TP+TN+FP+FN} \end{array}$$

(10) $$\begin{array}{l} SE=\frac{TP}{TP+FN} \end{array}$$

(11) $$\begin{array}{l} SP=\frac{TN}{TN+FP} \end{array}$$

where TP denotes the number of predicted vessel pixels inside the correct vessel segmentation of ground-truth and FN denotes the number of misclassified vessel pixels as background pixels. In a similar way, TN denotes the count of predicted background pixels inside the correct vessel segmentation of ground-truth and FP denotes the count of misclassified the background pixels as vessel pixels. In addition, the AUC can measure the performance of our method without being affected by the unbalanced classes.

### Ablation Studies

#### Ablation Study for Pre-processing Strategy

The effect of image pre-processing strategies can be demonstrated in this subsection. It is crucial for the image pre-processing to provide the accurate segmentation of the coronary arteries in X-ray angiograms. We implemented the following ablation experiments to evaluate the contributions of the pre-processing on DCA1 dataset, and Tables 1, 2 illustrate the results. We processed the coronary angiograms images before feeding them to our network with three pre-processing strategies respectively: without any pre-processing work, top-hat transforms, and CLAHE strategies. Figure 3 shows the coronary angiograms after pre-processing with these different strategies.

**Table 1:** Ablation study for pre-processing strategy on DCA1 dataset.

**Top-hat**	**CLAHE**	**SE**	**SP**	**ACC**	**AUC**
		0.8436	0.9789	0.9710	0.9860
✓		0.8793	0.9772	0.9714	0.9853
	✓	0.8755	0.9779	0.9719	0.9873

*DCA1, Database X-ray coronary angiograms; SE, sensitivity; SP, specificity; ACC, accuracy; AUC, area under curve*.

**Table 2:** Ablation study for pre-processing strategy on CCA dataset.

**Top-hat**	**CLAHE**	**SE**	**SP**	**ACC**	**AUC**
		0.9089	0.9717	0.9677	0.9890
✓		0.9072	0.9727	0.9677	0.9857
	✓	0.8798	0.9723	0.9664	0.9824

*CCA, the clinical coronary angiograms dataset; SE, sensitivity; SP, specificity; ACC, accuracy; AUC, area under curve*.

As shown in Table 1, we can deduce the conclusion that both the pre-processing strategies could improve the performance of segmentation in our work. Moreover, the network achieves the best performance after using CLAHE strategy to preprocess DCA1 dataset. The indicators SE, ACC, and AUC are all improved to varying degrees, while SP only decreases by 0.1%. The results in Table 2 demonstrate the impact of the two pre-process strategies on the CCA dataset. As can be seen from Table 2, the top-hat transform strategy is more suitable for the CCA dataset than the CLAHE strategy. In conclusion, we employ the CLAHE strategy when we pre-process the DCA1 dataset, while we use the top-hat transform strategy to preprocess CCA dataset in the following experiments.

#### Ablation Study for Expansion Factor *t*

We employed the bottleneck residual block as the main component of our network to reduce the parameters and computation so that our network becomes lightweight. The key to reducing parameters in the block is the depthwise separable convolution, which performs convolution on each channel separately and the channel dimension is constant. However, the convolution operation cannot extract enough information in low dimension. Hu et al. (23) addressed this problem by adding an expansion layer to expand the channels to a high dimension. We can tune the expansion factor *t* to achieve an optimal balance between accuracy and performance of our network. In this section, we performed four experiments to obtain a suitable expansion factor by trade-off.

Table 3 shows the different performances between the four expansion factors. With the increase in the expansion factor *t*, the increase in evaluation indicators is not significant. However, the number of parameters is greatly increased, thus the computational cost and running time are also increased. In order to balance the accuracy and performance, we assigned *t* to be 2 in the following experiments. From Table 3, we can find that the number of parameters is only 0.65 M when the expansion factor *t* = 2.

**Table 3:** Ablation study for expansion factor *t*.

***t***	**SE**	**SP**	**ACC**	**AUC**	**Params**
2	0.8755	0.9779	0.9719	0.9873	0.65 M
4	0.8904	0.9762	0.9712	0.9906	1.13 M
6	0.8915	0.9767	0.9717	0.9908	1.61 M
8	0.8882	0.9765	0.9713	0.9904	2.09 M

*SE, sensitivity; SP, specificity; ACC, accuracy; AUC, area under curve; Params, total params of our network; t, expansion factor; M, million*.

#### Ablation Study for Patch Attention Module

We developed the patch attention module (PAM) to model spatial long-range dependencies and make the model lightweight in our work. We performed two experiments to explore the effect of PAM: without PAM and with PAM embedded in the plain network (BRU-Net). Moreover, two comparative experiments are conducted on different patch sizes of PAM to analyze the impact of patch size on performance. We also used some of the above conclusions, which contain the combined pre-processing strategy, with the expansion factor 2. DCA1 dataset is used to verify the effectiveness of PAM, and the patch size is set to 2 × 2 in default.

**Figure 9:**
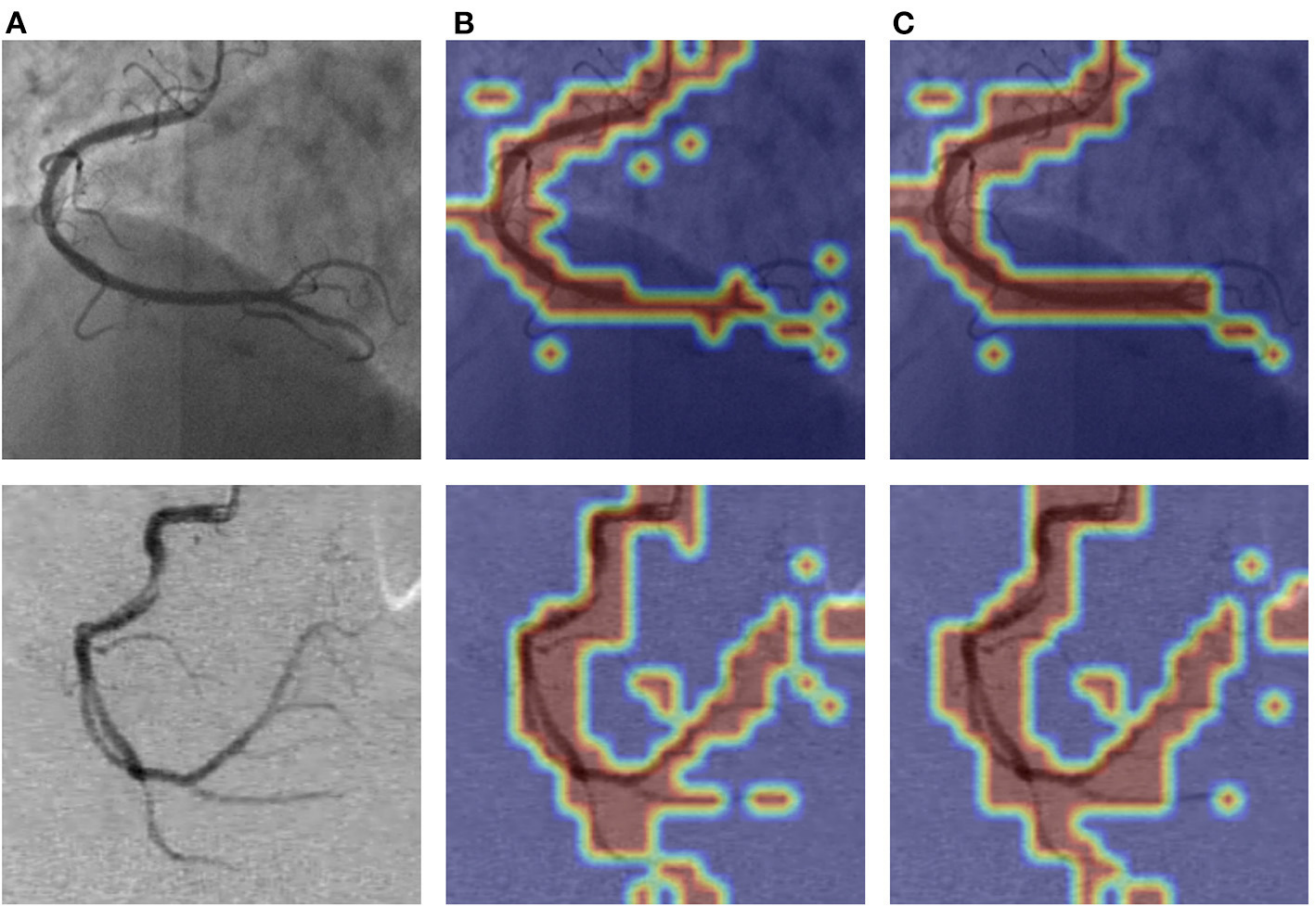
The visualization of PAM of the attention layer our network learned. The images in **(A)** column are the original angiograms, the images in **(B)** are the heatmaps after the encoder module without PAM, and the image in **(C)** are the attention heatmaps with PAM. The higher the frequency of the vessel regions, the brighter the color.

As shown in Figure 9, we visualized the output of the last layer in the encoder module. Obviously, the network with PAM focuses more attention on the vessel region, while the network without PAM lacks concentration on the region. As is seen from the results in Table 4, the network with PAM outperforms the one without PAM in the first three indicators. Table 5 shows the results of different patch sizes. A large patch size means fewer patches and lower computation, but it can be seen from Table 5 that SE indicators have decreased obviously, while the other three indicators do not change significantly. The SE indicator represents the condition of the vessel pixels the network correctly segments. Moreover, the vessel region is a very small part of the angiogram image. Thus, we set the patch size to 2 × 2 by trade-off.

**Table 4:** Ablation study for patch attention module (PAM).

**PAM**	**SE**	**SP**	**ACC**	**AUC**
	0.8755	0.9779	0.9719	0.9873
✓	0.8882	0.9774	0.9722	0.9889

*PAM, patch attention module; SE, sensitivity; SP, specificity; ACC, accuracy; AUC, area under curve*.

**Table 5:** Ablation study for patch size of PAM.

**Patch size**	**SE**	**SP**	**ACC**	**AUC**
2 × 2	0.8882	0.9774	0.9722	0.9889
4 × 4	0.8813	0.9778	0.9721	0.9901
6 × 6	0.8799	0.9779	0.9722	0.9874

*SE, sensitivity; SP, specificity; ACC, accuracy; AUC, area under curve; M, million*.

#### Ablation Study for SE Block

We investigated the effect of the squeeze and excitation (SE) block combined with the plain network on the performance in this section. We use BRU-Net as the backbone architecture and DCA1 dataset with pre-processing strategies. We utilized the recalibration of the channel-wise feature (23) and the lightweight of the SE blocks to improve the representational capacity of our network without adding too many parameters and computation. In this section, we conducted ablation experiments to verify the positive contribution of SE blocks to our network.

Table 6 shows the comparison results of the performance between the backbone network and the network with SE blocks. Specifically, the evaluation indicators of the network with SE blocks are more competitive than the backbone network. It can be concluded that the SE blocks are significant to improve the performance of our network. Moreover, we can see the fact that the network with SE blocks does not bring too many parameters. From Table 6, only 0.02 M parameters are brought in extra.

**Table 6:** Ablation study for squeeze and excitation (SE) block.

**SE block**	**SE**	**SP**	**ACC**	**AUC**	**Params**
	0.8755	0.9779	0.9719	0.9873	0.65 M
✓	0.8738	0.9785	0.9723	0.9898	0.67 M

*SE block, squeeze and excitation block; SE, sensitivity; SP, specificity; ACC, accuracy; AUC, area under curve; M, million*.

#### Experiments on the Integration of All Modules Above

In this section, we performed various experiments to verify the impact of using PAM and SE blocks together on the performance of our network. The experimental settings are similar to the above, and we employed the DCA1 dataset with the combined pre-processing strategy, the patch size was set to 2 × 2, and we assigned the expansion factor t of the bottleneck residual blocks to be 2.

To start with, we completed the experiments on the backbone network. Furthermore, we embedded the PAM into the backbone network to investigate the positive contribution of PAM, and then, we observed the capabilities that the SE blocks bring to the backbone network. Finally, we embedded both PAM and SE blocks into the backbone network to survey the benefit of performance while the two modules were used simultaneously. All the experiments' results in this section are illustrated in Table 7, and it is intuitive that both PAM and SE blocks are helpful for the coronary arteries' segmentation task. The two modules model the dependencies between the patches and channels respectively to enhance the representational capacity of the network. It has been proved that the performance of the network has been improved by the addition of PAM and SE blocks. In the next section, we employed both PAM and SE blocks in our experiments.

**Table 7:** Ablation study for PAM and SE block.

**PAM**	**SE block**	**SE**	**SP**	**ACC**	**AUC**	**Params**
		0.8755	0.9779	0.9719	0.9873	0.65 M
✓		0.8882	0.9774	0.9722	0.9889	0.73 M
	✓	0.8738	0.9785	0.9723	0.9898	0.67 M
✓	✓	0.8770	0.9789	0.9729	0.9910	0.75 M

*PAM, patch attention module; SE block, squeeze and excitation block; SE, sensitivity; SP, specificity; ACC, accuracy; AUC, area under curve; M, million*.

#### Experiments on the Generalization Ability of Our Network

In this section, we conducted the experiments on both the DCA1 dataset and the clinical coronary angiograms (CCA) dataset to investigate the generalization ability (57) of the proposed network. Firstly, we train our network on DCA1 and test the network on the DCA1 dataset. In addition, we used the trained model to predict the test dataset which is from the clinical dataset (CCA). Both the two test datasets are all unseen by our network before the test experiments.

**Figure 10:**
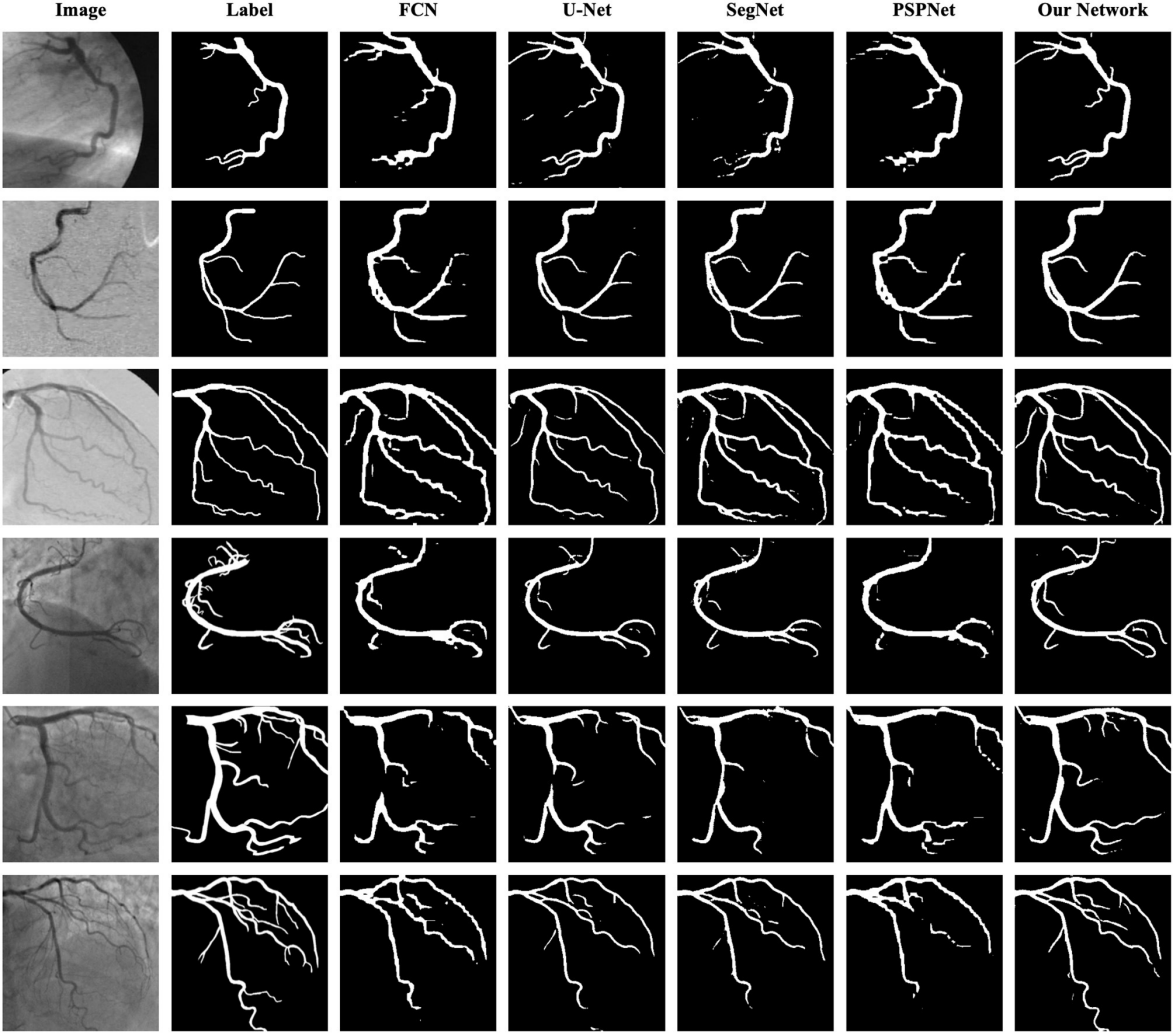
Segmentation results of FCN, U-Net, SegNet, PSPNet, and the proposed network. The images in the first column are the original angiograms, while the corresponding labels are in the second column. The images in the first three rows are from DCA1, the same dataset as the training set, while the images in the last three rows are from CCA.

As shown in Table 8 the training data are from DCA1 dataset, while the test data are from DCA1 and CCA dataset, respectively. From the results, we can see that the proposed network is suitable for the two coronary angiograms datasets, although the differences between the two datasets are quite obvious. In addition, we can find that our network performs equally excellently, even though the training and test data come from different datasets. The experiments above could be enough to prove that our network has a strong generalization ability. The last three rows of Figure 10 illustrate the prediction masks of each network, reflecting the generalization of each network. Compared with the prediction results of other networks, our network can predict the thin vessel regions well.

**Table 8:** Results of experiments on the generalization ability.

**Train data**	**Test data**		**SE**	**SP**	**ACC**	**AUC**
**DCA1**	**DCA1**	**CCA**				
✓	✓		0.8770	0.9789	0.9729	0.9910
✓		✓	0.7822	0.9828	0.9702	0.9808

*DCA1, Database X-ray Coronary Angiograms; CCA, the clinical coronary angiograms dataset; SE, sensitivity; SP, specificity; ACC, accuracy; AUC, area under curve*.

#### Comparative Experiments With Popular Segmentation Networks

As is known to all, plentiful excellent networks have been developed in the field of semantic segmentation, such as FCN (58), U-Net (27), SegNet (59), and PSPNet (60). To highlight the effectiveness of our network, we compared our method and its results with the approaches mentioned above. For the sake of fairness, we conducted these experiments by using the same preprocess strategies and the same loss function.

**Figure 11:**
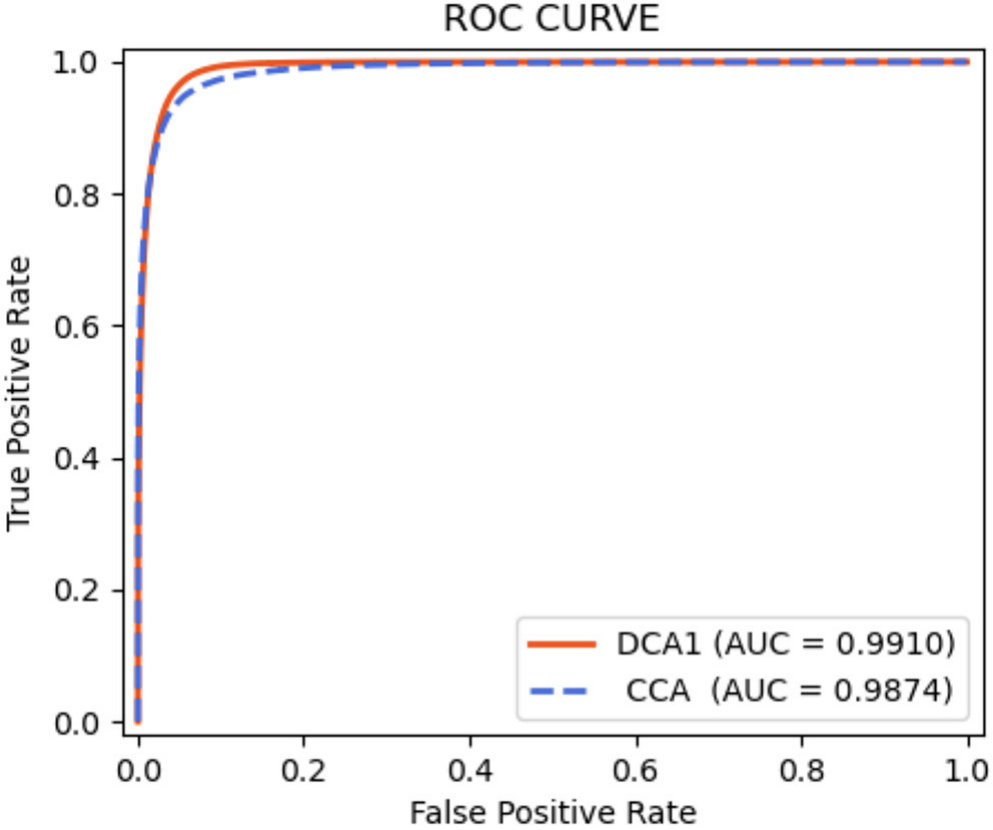
ROC curves for DCA1 and CCA datasets.

As shown in Table 9, the proposed network achieves more excellent performance in both DCA1 and CCA datasets than the other networks. Specifically, the proposed network obtains the best scores of SP, ACC, and AUC indicators, while only U-Net outperforms our network in SE indicator. It is worth noting that the number of parameters on our network is only 0.75 M, while the quantities of parameters on the other network are 20–60 times than ours. Figure 11 illustrates the ROC curve of the proposed network on DCA1 and CCA datasets. The larger the area under the ROC curve is the more accurate the inference of the network is. Figure 11 shows that our network can accurately segment blood vessels on two different datasets, while the segmentation result on DCA1 datasets is more accurate because the background of the coronary arteries in DCA1 are relatively straightforward. In addition, Figure 10 demonstrates the segmentation examples of all these networks. In the first three rows of Figure 10, the coronary arteries are from the same dataset as the training set, while the coronary arteries in the last three rows are not from the same dataset as the training set. From these examples, we can find that our network is better for the segmentation of thin blood vessels and does not identify the background regions as vessel regions like other methods, and the last three rows demonstrate that our network is more generalized.

**Table 9:** Results of the comparative experiments with popular segmentation networks.

**Method**	**Params**	**DCA1**				**CCA**			
		**SE**	**SP**	**ACC**	**AUC**	**SE**	**SP**	**ACC**	**AUC**
FCN	15.11M	0.8656	0.9701	0.9640	0.9863	0.8721	0.9557	0.9505	0.9772
U-Net	31.04M	0.8811	0.9762	0.9707	0.9900	0.9170	0.9559	0.9534	0.9850
SegNet	29.44M	0.8513	0.9776	0.9702	0.9878	0.8251	0.9719	0.9627	0.9765
PSPNet	46.58M	0.8500	0.9738	0.9666	0.9865	0.8666	0.9510	0.9457	0.9724
Proposed network	0.75M	0.8770	0.9789	0.9729	0.9910	0.8982	0.9706	0.9660	0.9874

*DCA1, database X-ray coronary angiograms; CCA, the clinical coronary angiograms dataset; SE block, squeeze and excitation block; SE, sensitivity; SP, specificity; ACC, accuracy; AUC, area under curve; M, million*.

## Discussion

In this study, we propose a lightweight network (BRU-Net) to automatically segment coronary arteries in X-ray angiograms that enables the method to be deployed on inexpensive mobile devices. It can help to realize the assistant diagnosis of CAD to become widespread in the regions where there is a lack of medical resources.

To overcome the above challenges, we mainly undertake the following efforts: accurate segmentation of coronary arteries on low quality angiographic images and making our segmentation network as lightweight as possible. Confronting the key problems, we propose the corresponding approaches. In addition, we employ the mixed loss function of weighted cross entropy loss function and dice loss to relieve the heavily unbalanced classes of coronary angiography images during the training procedure. Moreover, we have established a small database of coronary angiography to model and test our network.

For the low quality of coronary arteries, we employ two pre-processing strategies, Top-hat transform and contrast-limited adaptive histogram equalization (CLAHE) methods, to enhance the angiograms. Tables 1, 2 demonstrate that the employed pre-processing strategies can ease the problem of low quality. To make our network as lightweight as possible, we adopt bottleneck residual blocks to replace the internal components in the encoder and decoder of traditional U-Net to reduce the parameters and computation. We also compare the size of the expansion factor *t* to further optimize the balance between the number of parameters and network performance. Table 3 shows the different effects of different expansion factors on segmentation results. To further improve the segmentation performance of the lightweight network, we utilize two lightweight attention modules with a few additional parameters. We develop a novel attention module, named the patch attention module (PAM), to model spatial long-range dependencies and make the model lightweight, while we employ the squeeze and excitation (SE) block in each bottleneck residual block to capture the correlation between channels in the feature maps. The inspiration for PAM comes from the position attention module (24), while PAM is much lighter than it (24). The space complexity of PAM in the proposed network is $\frac{1}{2^{4}}$ than that of the position attention module (24). From Figure 9, we can find that the network with PAM focuses more attention on the vessel region, and Table 7 shows that the two addition modules can promote the proposed network to obtain better segmentation results. As shown in Table 9, the number of parameters is only 0.75 M in the proposed network, which is just a fraction of those in other networks, with almost the same segmentation performance. Furthermore, we prove the generalization and robustness of our network by using training sets and test sets from different datasets. From Table 8, we can find that the proposed network is suitable for the two coronary angiograms datasets, although the differences between the two datasets are quite obvious. Some examples of segmentation results between our network and other networks are characterized in Figure 10. As illustrated in Figure 10, our network can segment thin and irregular vessels well from low-quality angiographic images. Also, from the last three rows of Figure 10, the proposed network can segment the coronary angiograms of different datasets well.

From the above discussion, the proposed network can not only segment the coronary artery accurately but also be deployed in inexpensive mobile devices due to its characteristic lightweight to popularize assistant diagnosis of CAD and to provide convenience for experts. An accurate and readily available coronary artery segmentation can be frugal with specialists' time and provide a more effective diagnostic basis.

Our lightweight network achieves good results in coronary artery segmentation with a few parameters. However, the network sometimes segments the background of coronary angiography into vessels regions, while some tiny vessels are treated as the background. In addition, in the process of data annotation, some thin blood vessels are not marked, but they are segmented by our network, which also affected various indicators of the final segmentation results. The same situation occurs in the DCA1 dataset. We advocate establishing some larger and better coronary angiography databases to contribute to the auxiliary diagnosis of coronary artery disease.

## Conclusion

This study presents a novel and lightweight network for coronary arteries segmentation in X-ray angiograms. We adopt bottleneck residual blocks to replace the internal components in the traditional U-Net to make the network more lightweight. To make the segmentation of coronary arteries more accurate, we embedded the two attention modules to model long-range dependencies in spatial and channel dimensions. Comprehensive experimental analysis and ablation study on two X-ray coronary angiograms datasets demonstrate that our method is effective, robust, and lightweight enough. In the future, we will devote more energy to the research of more lightweight coronary artery segmentation networks with better segmentation performance to assist clinical diagnosis to help more patients with coronary artery disease.

## Data Availability Statement

The original contributions presented in the study are included in the article/supplementary material, further inquiries can be directed to the corresponding authors.

## Author Contributions

XT and HD: conceptualization. XT: methodology and writing—original draft preparation. XT, HD, XZ, XX, and DX: validation. XT and HD: investigation and writing—review and editing. XX, XZ, and DX: resources. XX and DX: data curation. XZ and XX: supervision. All authors have read and agreed to the published version of the manuscript.

## Funding

This work was supported by the Foundation of Shanghai Key Medical Specialty under Grant 2018-KY-04, the 111 Project under Grant B08004, and Scientific and Technological Project of Henan Province under Grant 222102210028.

## Conflict of Interest

The authors declare that the research was conducted in the absence of any commercial or financial relationships that could be construed as a potential conflict of interest.

## Publisher's Note

All claims expressed in this article are solely those of the authors and do not necessarily represent those of their affiliated organizations, or those of the publisher, the editors and the reviewers. Any product that may be evaluated in this article, or claim that may be made by its manufacturer, is not guaranteed or endorsed by the publisher.
